# Inflammatory events during murine squamous cell carcinoma development

**DOI:** 10.1186/1476-9255-9-46

**Published:** 2012-11-23

**Authors:** Thais Helena Gasparoto, Carine Ervolino de Oliveira, Luisa Thomazini de Freitas, Claudia Ramos Pinheiro, Rodrigo Nalio Ramos, André Luis da Silva, Gustavo Pompermaier Garlet, João Santana da Silva, Ana Paula Campanelli

**Affiliations:** 1 Department of Biological Sciences - Microbiology and Immunology, Bauru School of Dentistry, University of São Paulo, Bauru, SP, Brazil; 2 Department of Stomatology - Oral Pathology, Bauru School of Dentistry, University of São Paulo, Bauru, SP, Brazil; 3 Department of Biochemistry and Immunology, School of Medicine of Ribeirão Preto, University of São Paulo, Ribeirão Preto, SP, Brazil

**Keywords:** Elastase, Nitric oxide, Myeloperoxidase, Inflammatory cells, Cytokines

## Abstract

**Background:**

Squamous cell carcinoma (SCC) is one of the most common human cancers worldwide. In SCC, tumour development is accompanied by an immune response that leads to massive tumour infiltration by inflammatory cells, and consequently, local and systemic production of cytokines, chemokines and other mediators. Studies in both humans and animal models indicate that imbalances in these inflammatory mediators are associated with cancer development.

**Methods:**

We used a multistage model of SCC to examine the involvement of elastase (ELA), myeloperoxidase (MPO), nitric oxide (NO), cytokines (IL-6, IL-10, IL-13, IL-17, TGF-β and TNF-α), and neutrophils and macrophages in tumour development. ELA and MPO activity and NO, IL-10, IL −17, TNF-α and TGF-β levels were increased in the precancerous microenvironment.

**Results:**

ELA and MPO activity and NO, IL-10, IL −17, TNF-α and TGF-β levels were increased in the precancerous microenvironment. Significantly higher levels of IL-6 and lower levels of IL-10 were detected at 4 weeks following 7,12-Dimethylbenz(a)anthracene (DMBA) treatment. Similar levels of IL-13 were detected in the precancerous microenvironment compared with control tissue. We identified significant increases in the number of GR-1^+^ neutrophils and F4/80^+^/GR-1^-^ infiltrating cells in tissues at 4 and 8 weeks following treatment and a higher percentage of tumour-associated macrophages (TAM) expressing both GR-1 and F4/80, an activated phenotype, at 16 weeks. We found a significant correlation between levels of IL-10, IL-17, ELA, and activated TAMs and the lesions. Additionally, neutrophil infiltrate was positively correlated with MPO and NO levels in the lesions.

**Conclusion:**

Our results indicate an imbalance of inflammatory mediators in precancerous SCC caused by neutrophils and macrophages and culminating in pro-tumour local tissue alterations.

## Introduction

Inflammatory responses play decisive roles in different stages of tumour development, including initiation, promotion, progression, invasion, and metastasis. The tumour microenvironment, which is orchestrated by inflammatory cells, affects malignant cells through the production of cytokines, chemokines, growth factors, prostaglandins, reactive oxygen species (ROS) and nitric oxide (NO) [1-5]. Sub-lethal levels of ROS and NO, which are produced by activated neutrophils and macrophages, drive cancer development by inducing DNA damage [6-8]. They also stimulate cancer cell proliferation, assisting tumour establishment [5,9]. Myeloperoxidase (MPO), which is abundantly expressed in neutrophils and to a lesser extent in monocytes and certain type of macrophages [10], has been strongly correlated with different types of cancer progression due to its role in ROS generation [2,9,11]. Additionally, the proteolytic enzyme elastase (ELA) is also involved with carcinogenesis and metastasis through degradation of the extracellular matrix, facilitating cancer invasion [12,13].

Squamous cell carcinoma (SCC) is one of the most common cancers in humans and typically arises from mutated ectodermal or endodermal cells lining body cavities. While SCC can occur in a large number of tissues, cells in the skin are frequently associated with cellular abnormalities in the basal layer of the epidermis resulting from UV-damaged keratinocytes [14-16]. Although immunosuppression is currently considered to be a risk factor for SCC, inflammation is involved in SCC establishment, and UV light has been demonstrated to increase inflammatory infiltrates, which enhances skin tumour growth [17,18]. In this manner, CXCL8 has been suggested as an earlier biomarker for SCC [19] because this chemokine, one of the most important neutrophil chemotactic and activating factors, is related to angiogenesis, tumour growth and metastasis [20]. However, other cytokines and chemokines that coordinate leukocyte migration to inflammatory sites and cellular trafficking through the lymph nodes and the spleen have been associated with SCC development [20,21]. The two-stage 7,12-dimethylbenz(a)-anthracene (DMBA)/12-O-tetradecanoylphorbol-13-acetate (TPA) skin carcinogenesis model, which triggers the initiation and promotion steps, respectively, has been commonly used to mimic squamous cell carcinoma, allowing for the investigation of several aspects of SCC [22,23]. TPA/PMA tumour promotion is based on protein kinase C (PKC) activation culminating in the release of reactive oxygen species (ROS) [24,25].

Because inflammatory events have been implicated in carcinogenesis and neutrophil infiltration is correlated with some types of cancer metastasis [26,27], we used a multistage model of SCC to examine the involvement of ELA, MPO, NO, cytokines and inflammatory cells in tumour development.

## Methods

### Mice

Eight-week-old female BALB/c mice were purchased from the Bauru School of Dentistry, University of São Paulo. Each mouse was housed in an isolated cage. Food and water were provided ad libitum. The mice were maintained on a 12-h light/12-h dark photocycle in a controlled temperature environment and were quarantined for a minimum of 1 week before treatment. Groups of mice were randomly euthanised between 4 weeks and 16 weeks following 7,12-dimethylbenz-anthracene (DMBA) (Sigma-Aldrich®, St. Louis, MO, USA) application. A total of 36 mice were used in the study. All animal experiments were approved by the Animal Research Ethics Committee of the Bauru School of Dentistry, University of São Paulo.

### DMBA/PMA-induced skin carcinogenesis initiation-promotion experiments

The experimental group received DMBA and 12-O-tetradecanoyl-phorbol-13-acetate (TPA) (Sigma-Aldrich®) as follows. Eight-week-old female mice were divided into 3 groups of three mice (at 4^th^, 8^th^ and 16^th^ weeks) each and were topically treated with four doses of DMBA (25 μg in 200 μl of acetone) and biweekly doses of TPA (200 μl of a 10^–4^ M solution in acetone) for 16 weeks. The experiment was performed 3 times. Papilloma and carcinoma development were monitored every three days throughout the experiment. Papillomas were characterised by folded epidermal hyperplasia protruding from the skin surface, and carcinomas were characterised as endophytic tumours presenting as plaques with an ulcerated surface. Experimental animals were cared for in accordance with institutional guidelines. Untreated mice were used as the control group. Samples were collected at different time points after initiation and were processed as described below. Lesions were initially identified macroscopically and subsequently identified through histological diagnosis.

### Measurement of tumour growth

Skin tumours were measured using a precision calliper allowing discrimination to size modifications >0.1 mm. Tumour volumes were measured the first day of treatment and every week until the day that they were humanely killed and the lesions were measured according to followed: volume = 0.4 ab^2^, where a and b are the larger and smaller diameters, respectively [28].

### Histological analysis

Tissue samples were collected from tumour sites and fixed with 10% (v/v) formalin for 6 hours at room temperature. The tissues were subsequently dehydrated in ethyl alcohol followed by washes in xylol and were then embedded in paraffin. Each sample was sectioned into 5- to 7-μm-thick slices that were dried onto slides and stained with hematoxylin and eosin.

### Isolation of leukocytes

To characterise the leukocytes present at the tumour site, biopsies of skin lesions from mice were collected and incubated for 1 h at 37°C in RPMI 1640 medium containing 50 μg/mL of a collagenase CI enzyme blend (Boehringer Ingelheim Chemicals, Normandy Drive Petersburg, VA, USA). The tissues were subsequently dissociated for 4 min in RPMI 1640 (GIBCO®, Life Technologies, Staley Road Grand Island, NY, USA) with 10% bovine foetal serum (GIBCO®, Life Technologies) and 0.05% DNase (Sigma-Aldrich®) using a Medimachine (BD Biosciences, Qume Drive San Jose, CA, USA) cytometry sample preparation system, according to the manufacturer’s instructions. The tissue homogenates were filtered using a 30-μm cell strainer (Falcon; BD Biosciences). Leukocyte viability was evaluated by Trypan blue exclusion, and these cells were subsequently used for cell activation and immunolabelling assays.

### Antibodies (Abs) and flow cytometry analysis

For immunostaining, PE- and FITC-conjugated Abs directed against CD11b (17A2), LY6G/GR-1^+^ (H129.19), F4/80 (6F12) and the respective goat and rat isotype controls were used (BD Biosciences). Intracellular IL-17 (BD Biosciences) in leukocytes obtained from lesions and lymph nodes was detected using Cytofix/Cytoperm and Perm/Wash buffer from BD Biosciences, according to the manufacturer’s instructions. Briefly, the cells were labelled with Abs directed against the cell surface antigens. Following surface staining, the cells were fixed, permeabilised, and stained with PE-labelled anti-mouse IL-17 (MACS Miltenyi Biotech, Miltenyi Biotec GmbH, Bergisch Gladbach, Germany) or the isotype control. The samples were acquired on a FACSort flow cytometer, and the data were analysed using CellQuest software (BD Biosciences).

### Immunofluorescence analysis and confocal microscopy

Slides for double immunofluorescence staining were post-fixed with 4% paraformaldehyde and blocked with protein-block assay diluent (BD Company). After washing with PBS, the slides were incubated with the primary antibody, washed again, and incubated with the appropriate fluorochrome-conjugated (Texas Red or FITC) secondary antibodies. After washing, the slides were mounted using mounting medium with DAPI (Vector Laboratories®, Burlingame, CA, USA) to stain the nucleus and were then analysed by confocal microscopy. Images were captured with a Leica TCS SPE confocal laser system equipped with a 63 oil-immersion plan apochromatic objective (1.3 CS) with differential interference contrast. LAS AF 2.5.1 software was used for image acquisition.

### Cytokine assays

The tumour sample supernatants were obtained by disaggregation through treatment with RPMI 1640 medium containing 0.25% collagenase (Worthington Biochemical Corporation, Lakewood, NJ, USA) and were frozen at −80°C until analysis. The total protein concentration was measured using a Quick StartTM Bradford Protein assay kit (Bio-Rad, CA, USA). TNF-α, IL-6, IL-10 and TGF-β levels in the samples were quantified using a quantitative sandwich enzyme-linked immunosorbent assay (ELISA) that employed commercial capture and biotinylated detection antibodies (BD Pharmingen Corp., San Diego, CA), and the respective recombinant mouse cytokines (diluted in PBS) as standards according to the manufacturer’s instructions. IL-13 and IL-17 levels were determined using an eBioscience kit (eBioscience®, San Diego, CA, USA) according to the manufacturer’s instructions. The concentration of each cytokine was dosed as pg/mL, and the results were normalised and expressed as mg/protein.

### Myeloperoxidase (MPO) and elastase (ELA) activities

MPO and ELA activities in the samples were assessed after obtaining tissue supernatants by disaggregation through treatment with RPMI 1640 (Gibco) medium containing 0.25% collagenase (Worthington Biochemical Corporation) as described previously [29].

### Nitric oxide production

To detect NO in lesions or skin samples, nitrite (NO-2) production was measured in the supernatant samples using the Griess method [29]. Briefly, 50 μL of supernatant samples were incubated with an equal volume of Griess reagent at room temperature. The absorbance was measured on a plate scanner (Spectra Max 250; Molecular Devices, Sunnywale, California, USA) at 540 nm. The NO-2 concentration was determined using a standard curve for NaNO_2_ at a concentration range from 1 to 200 μM.

### Statistical analysis

The results are expressed as the mean ± SD, and statistical analysis was performed using unpaired Student’s t-tests to compare each experimental group with the control group and a one-way ANOVA followed by Tukey’s test to compare all groups (GraphPad software 4). p ≤ 0.05 was considered to indicate statistical significance.

## Results

The appearance of chemically induced papillomas is accompanied by increased neutrophil infiltration

**Figure 1:**
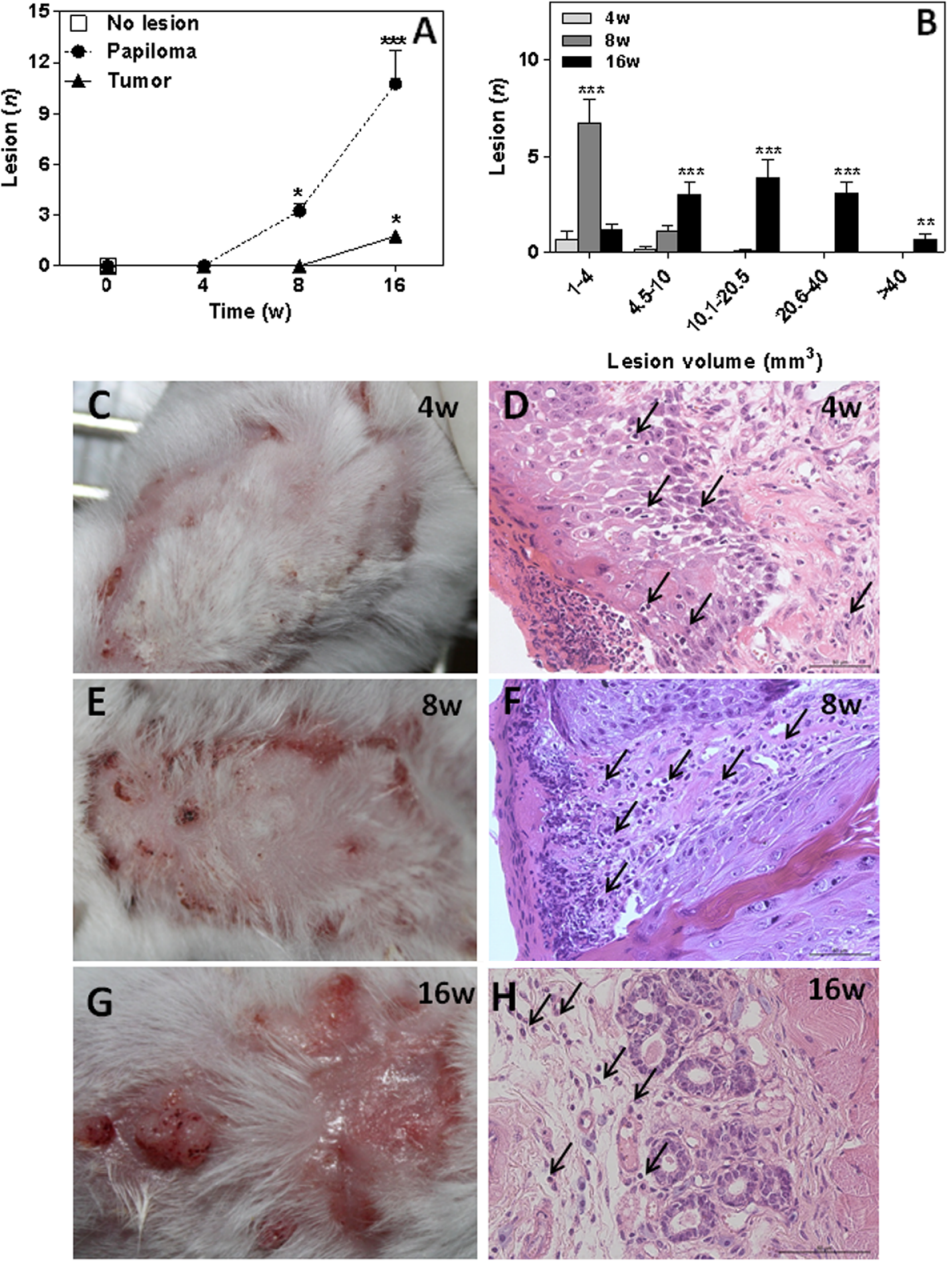
**Squamous cell carcinoma induced by DMBA/TPA in mice.** SCC mice were treated according to a chemical carcinogenic protocol using DMBA and TPA for 16 weeks. Papilloma incidence (**A**) and tumor volume (**B**) were determined in SCC mice. Each value represents mean ± SEM of 9 different mice. *P<0.05, **P<0.01 and *** P< 0.001. Panels **C**, **E** and **G** are representative photomicrographs of dorsal tissue from SCC mice. Haematoxylin and eosin staining of skin tissue sections from BALB/c mice 4(**D**), 8(**F**) and 16 (**H**) weeks after chemical carcinogenesis. Data are from one experiment that is representative of three independent experiments (n = 9 mice per group). Arrows indicate inflammatory cells.

Papillomas were found in 100% of DMBA/TPA-treatment mice seven weeks after carcinogenic induction (data not shown). The greatest number of papillomas was found at 16 weeks (10.7 ± 2 lesions) (Figure 1A and 1G). Lesions found at this time were significantly more extensive (since 4.5 until >40 mm) than those found during the 4^th^ and 8^th^ weeks (Figure 1B). Histological analysis revealed polymorphonuclear cells in the superficial layers of the epithelium at 4 weeks (Figure 1D), with pronounced inflammatory cell presence and intense epithelial cell mitotic activity at 8 weeks (Figure 1F). Intense inflammatory infiltrate and mitotic activity and epithelial islet formation were observed at 16 weeks after DMBA/TPA treatment (Figure 1H). We identified polymorphonuclear and mononuclear inflammatory cells in different layers of the skin tissue after DMBA treatment (Figure 1D, 1F and 1H).

Inflammatory mediators levels during the establishment of SCC.

**Figure 2:**
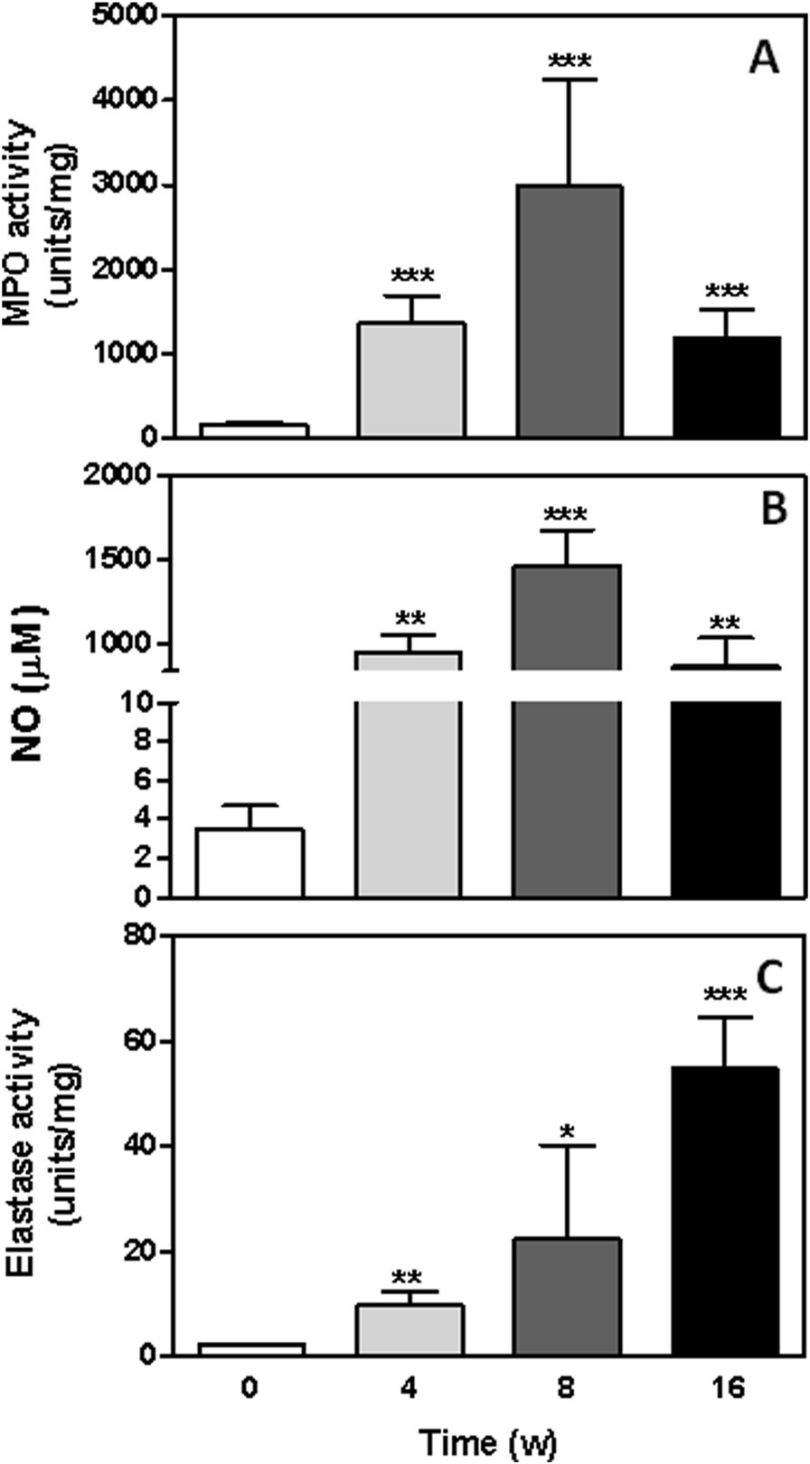
**MPO, NO and ELA levels in the tumour microenvironment.** MPO (**A**), NO (**B**) and ELA (**C**) levels were analyzed in the tumor and control untreated tissue as described in the methods and materials section. Results are expressed as the mean ± SEM from each individual mouse analyzed. *P<0.05, **P<0.01 and *** P< 0.001.

Because tissue damage triggered by inflammatory mediators is associated with cancer establishment, we evaluated MPO and ELA activity in the chemically treated tissue samples (Figure 2). The results showed increased MPO activity at 4 weeks (1348 ± 334.7 units/mg), 8 weeks (2975 ± 1231 units/mg) and 16 weeks (1187 ±3 21.8 units/mg) following DMBA/TPA treatment in comparison with control mice (159.9 ± 12.3 units/mg) (Figure 2A).

NO levels in the tissue samples were significantly higher after chemical treatment compared to the control group (Figure 2B). Interestingly, the highest levels of NO were detected in the 8-week group (1460 ± 215.1μM) (Figure 2B), which was also verified by MPO activity (Figure 2A).

ELA activity increased as a function of the time of treatment. The 4-week (9.8 ± 2.4 units/mg), 8-week (22.4 ± 17.2 units/mg) and 16-week (54.6 ± 9.9 units/mg) groups all had ELA activities that were significantly higher than that of the control group (2.19±0.2 units/mg) (Figure 2C).

### Cytokine levels in the tumour microenvironment during the establishment of SCC

**Figure 3:**
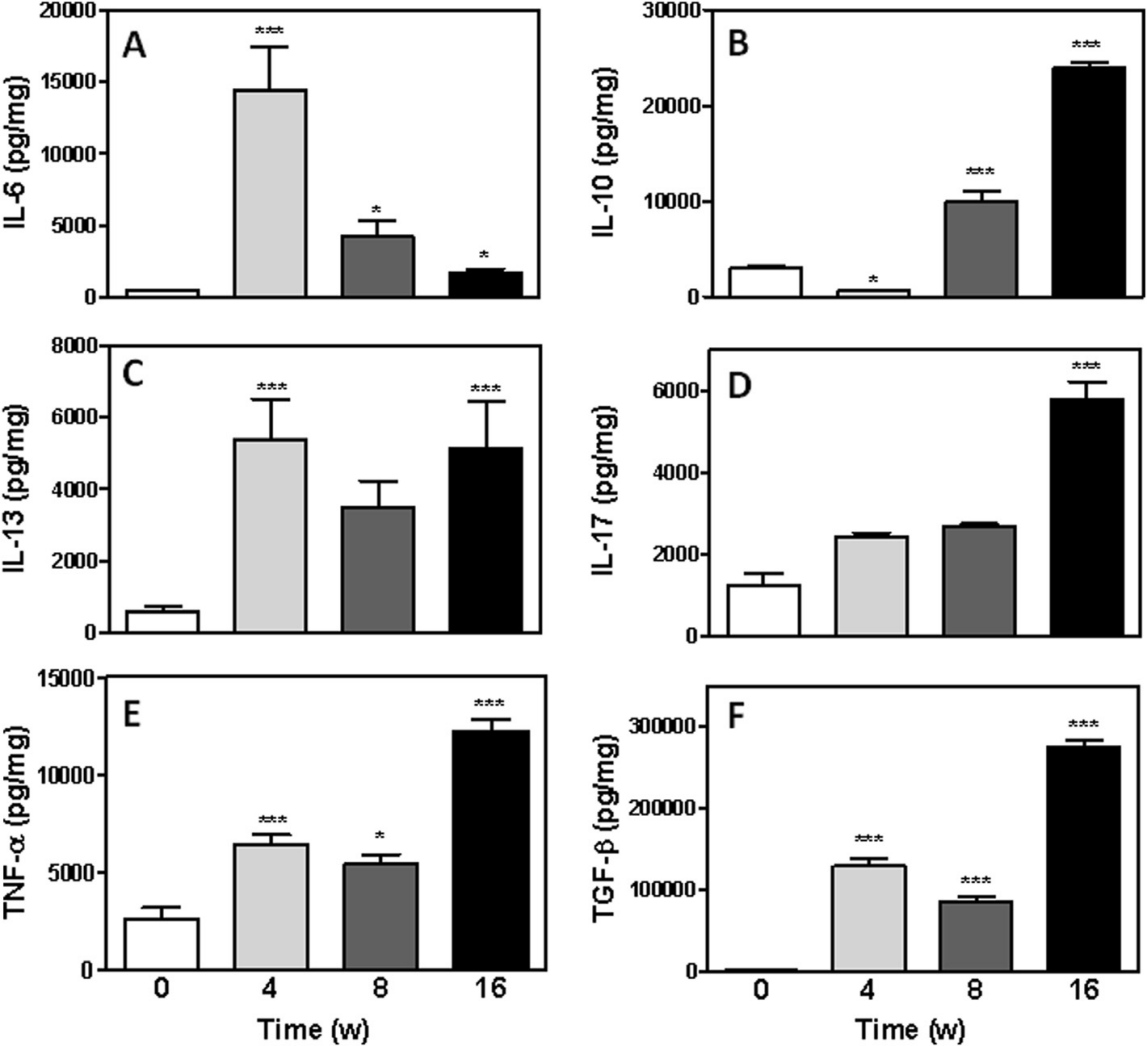
**Cytokine levels in the tumour microenvironment.** IL-6 (**A**), IL-10 (**B**), IL-13 (**C**), IL-17 (**D**), TNF-α (**E**) and TGF-β (**F**) levels were analyzed by ELISA. Results are expressed as the mean ± SEM from each individual mouse analyzed (n=9 mice per group). *P<0.05, **P<0.01 and *** P < 0.001.

To evaluate cytokine expression during the development and establishment of experimental SCC, we analysed IL-6, IL-10, IL-13, IL-17, TNF-α and TGF-β levels in the lesion tissues (Figure 3). At 16 weeks following DMBA treatment, levels of all of these cytokines with the exception of IL-6 had significantly increased in the treated tissues compared with control skin (Figure 3A- 3F). Among the treated groups, significantly higher levels of IL-10, IL-17, TNF-α and TGF-β were detected in the 16 week group compared with the 4 and 8 week groups (Figures 3B, 3D, 3E and 3F). Although IL-13, TNF-α and TGF-β levels increased in all groups compared with control samples (Figures 3C, 3E and 3F), IL-10 levels decreased at 4 weeks after DMBA treatment (620.8 ± 68 pg/mg) and increased to levels higher than that of the control group at 8 weeks (9876 ± 1120 pg/mg) (Figure 3B). The highest levels of IL-6 were detected at 4 weeks (14403 ± 3026 pg/mg) compared to the 8 week (4193 ± 1065 pg/mg), 16 week (1673 ± 309.8 pg/mg) and control groups (474.9 ± 11.1 pg/mg) (Figure 3A).

In agreement with these data, a significant increase in cytokine levels was detected during SCC development compared with the untreated group (week 0). We verified that the highest levels of the cytokines IL-10, IL-17, TNF-α and TGF-β were present at 16 weeks (Figure 3).

### Determination of macrophages and neutrophils infiltrating squamous cell carcinoma lesions

**Figure 4:**
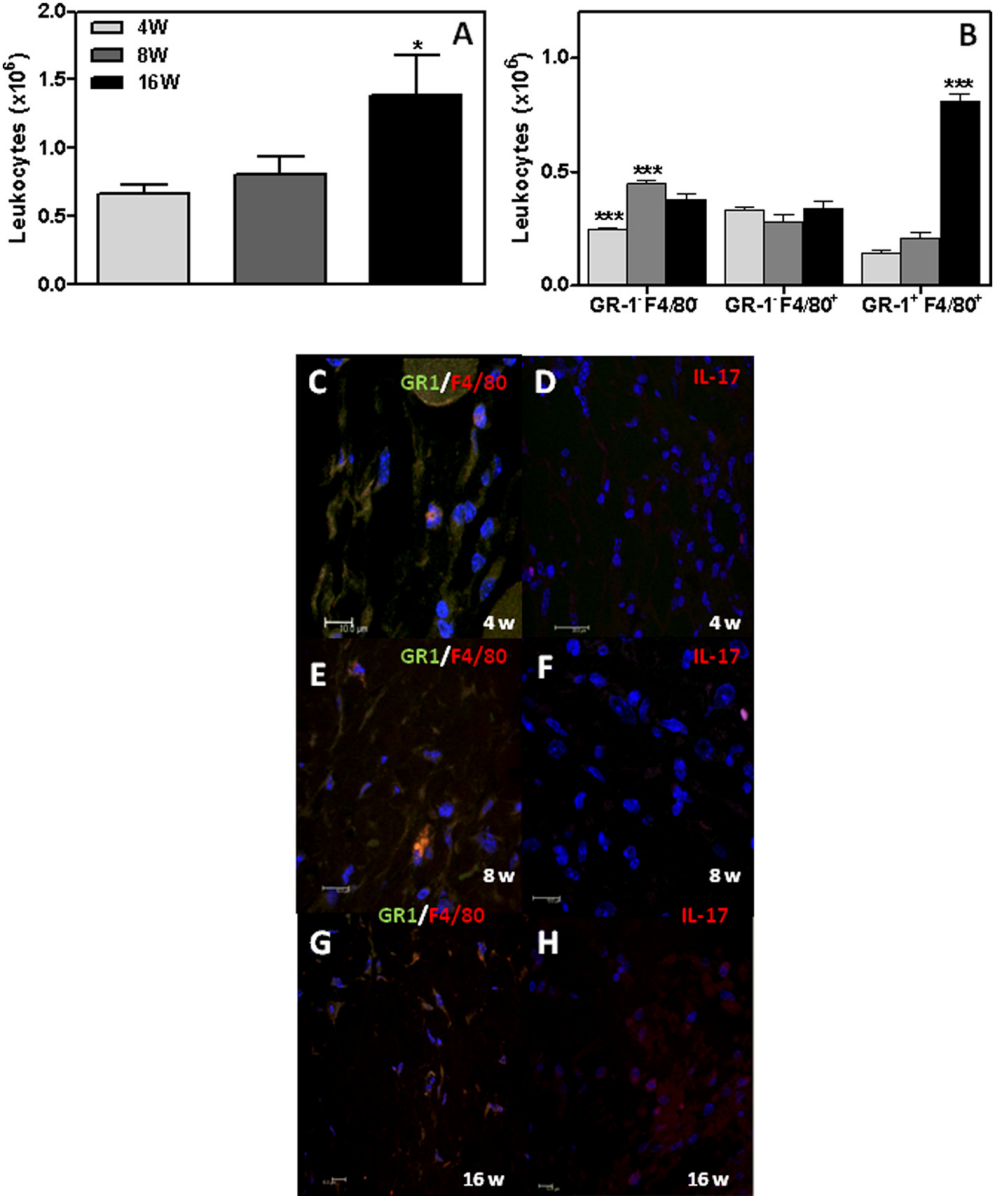
**Inflammatory infiltrates in mouse squamous cell carcinoma.** The total number of leukocytes (**A**) and the number of cells expressing GR1 and F4/80 (**B**) were determined during the 4^th^ (4W), 8^th^ (8W) and 16^th^ (16W) weeks after DMBA protocol. Results are expressed as the mean ± SEM from each individual mouse analyzed (n=9 mice per group). *P< 0.05 and *** P< 0.001. Representative photomicrograph of GR1^+^ (green), F4/80^+^ (red) and IL-17^+^ cells (red) infiltrating tumour lesions. Representative tumour is shown. Blue, DAPI.

To determine if the increased cytokine levels recruited increased numbers of inflammatory cells, we evaluated the number of leukocytes in the lesions (Figure 4A). As expected, the number of leukocytes infiltrating the lesions increased over time, increasing from 0.7 ± 0.06x10^6^ in the 4^th^ week to 0.9 ± 0.13x10^6^ in the 8^th^ week and 1.5 ± 0.3x10^6^ in the 16^th^ week (Figure 4A). However, significant differences were only detected between the 16-week group and the 4- and 8-week groups (p<0.05, Figure 4A).

We next analysed the inflammatory infiltrates, assessing the presence of neutrophils and macrophages in chemically treated tissues from the mice at 4, 8 and 16 weeks following DMBA treatment (Figure 4B- 4C). To determine the neutrophil and macrophage phenotypes present in the tissues infiltrates, the percentage of cells expressing GR-1 and F4/80 were evaluated by flow cytometry (Figure 4B). Neutrophils, as characterised by a GR-1^+^/F4/80^-^ phenotype, were increased at 4 (34.6 ± 2.5%) and 8 weeks (55.9 ± 3.2%) compared with 16 weeks (27.3 ± 3.8%). Macrophages were also present at higher percentages in the 4^th^ (47.7 ± 4.9%) and 8^th^ (35.6 ± 3.2%) week compared to the 16^th^ week (24.3 ± 6.1%) (Figure 4B). However, macrophages exhibiting an activated phenotype and characterised by expression of both GR-1 and F4/80 were present at a significantly higher concentration at 16 weeks (57.7 ± 0.9%) than at 4 (20.5 ± 5.7%) or 8 weeks (26.5 ± 5.9%) following DMBA/TPA treatment (Figure 4B). Representative photomicrographs show immunofluorescence staining for 4 weeks (Figure 4C-D), 8 weeks (Figure 4E-F) and 16 weeks (Figure 4 G-H) following DMBA application.

Although the milieu of cytokines and oxidative compounds might influence SCC establishment and progression, the lesions only showed correlation with ELA, IL-10 and IL-17 (Table 1).

**Table 1:** Correlation between lesions and inflammatory mediators during the chemical-induced squamous cell carcinoma development

	**Lesions**		
	**Correlation (r)**^**2**^	***P***	**Pearson r**
ELA	0.9761	0.0120*	0.9880
IL-10	0.9799	0.0101*	0.9899
IL-17	0.9309	0.0352*	0.9648
Neutrophils	0.0066	0.9186	0.08144
Macrophages	0.9846	0.0154*	0.9695

*Statistically significant difference comparing lesions appearance with elastase (ELA) activity, IL-10 and IL-17 levels, and neutrophils or macrophage infiltration.

Neutrophil tissue infiltration was positively and significantly correlated with MPO and NO levels in the epithelial tissues (Table 2). While we did not find any correlation between the presence of neutrophils and papillomas or tumour lesions, macrophages were positively and significantly correlated with both lesions (Tables 2 and s 3). In addition, macrophages were positively and significantly correlated with ELA activity and IL-10 and IL-17 levels in epithelial tissues between 0 and 16 weeks (Table 3).

**Table 2:** Correlation between neutrophils and inflammatory mediators during the chemical-induced squamous cell carcinoma development

	**Neutrophils**		
	**Correlation (r)**^**2**^	***P***	**Pearson r**
MPO	0.9211	0.0430*	0.9761
NO	0.9717	0.0142*	0.9858
ELA	0.0509	0.7743	0.2257
Papiloma	0.0123	0.8888	0.1112
Tumor	0.1436	0.8802	-0.1198

*Statistically significant difference comparing neutrophils infiltration with myeloperoxidase (MPO) activity, nitric oxide (NO) level, elastase (ELA) activity, and number of papilloma and tumor lesions.

**Table 3:** **Correlation between F4/80**^**+**^**GR1**^**+**^**macrophages and inflammatory mediators during chemical-induced squamous cell carcinoma development**

	**Macrophages**		
	**Correlation (r)**^**2**^	***P***	**Pearson r**
ELA	0.9895	0.0053**	0.9947
IL-10	0.9140	0.0439*	0.9561
IL-17	0.9915	0.0043**	0.9957
Papiloma	0.9665	0.0169*	0.9831
Tumor	0.9114	0.0453*	0.9547

*Statistically significant difference comparing macrophages GR1^+^F4/80^+^ infiltration with elastase (ELA) activity, IL-10 and IL-17 levels, and number of papilloma and tumor lesions.

## Discussion

Cancer is a complex, multistage process characterised by molecular alterations regulated by both genetic and epigenetic mechanisms [30]. Because DNA lesions and methylation states are influenced by oxidative species catalysed by MPO, it is logical to assume that an association exists between this enzyme and cancer initiation [30-32]. Polymorphisms in the MPO gene promoter region are associated with a reduced risk of cancer [33-35]. Here, we demonstrate the presence of neutrophils and activated macrophages during the development of chemically induced squamous cell carcinoma. This cell infiltration was accompanied by myeloperoxidase and elastase activity and the presence of nitric oxide. Both myeloperoxidase (MPO) and elastase (ELA) are enzymes that are abundantly secreted by activated neutrophils, a mechanism that helps these cells to defend against aggression [10,36]. MPO dimeric alpha-heme halo peroxidase present in azurophilic granules makes up approximately 5% of the dry weight of the neutrophil [37]. Although MPO is correlated with a better prognosis in different types of tumours such as breast cancer [34,38-41], the majority of studies have shown an important role for MPO in cancer progression [2,9,11,31]. It was shown that TPA-stimulated mouse neutrophils exhibit DNA damage resulting from hydrogen peroxide-induced breaks [42]. In support of this finding, we found MPO to be significantly more active in chemically treated mice than in control mice, and we found a positive correlation with neutrophil infiltration.

Both MPO and ELA appeared to contribute to tissue and extracellular matrix degradation, enhancing cancer development by destroying natural barriers against metastasis [43,44]. Several studies have also described elastinolytic enzyme production by human and rodent mammary tumour cells that facilitates their dissemination [10,45,46]. ROS-mediated oxidative tissue damage and ROS-mediated upregulation of the gene expression responsible for recruitment of inflammatory cells can both inhibit tumour growth and support the metastatic growth of tumour cells [5,24,25].

Although three types of ELA have been characterised in mammals, only neutrophil elastase (NE) is able to degrade insoluble elastin and hydrolyse other extramatrix proteins, including fibronectin, proteoglycans, and type IV collagen [13,47,48]. NE has also been shown to increase cancer cell malignancy through mechanisms that are still unclear [13]. We detected high ELA activity at 4 weeks after DMBA/TPA treatment that persisted until 16 weeks and increased as the lesions grew. It is possible that ELA sources such as macrophages, neutrophils, and cancer cells change during chemically induced SCC development. Our data showed that macrophage but not neutrophil infiltration was correlated with ELA activity in the lesions. This result should be further elucidated in the future.

Because our results indicated the involvement of inflammation during chemically induced SCC development, and a key molecular link between inflammation and tumour promotion and progression is the NF-kB signalling pathway, which is activated by many proinflammatory cytokines [49,50], we analysed cytokine production in the tumour microenvironment. IL-6 was significantly enhanced at 4 weeks after DBMA/TPA treatment, while IL-10 levels were lowest in these samples (Figure 4). IL-6 and TNF-α are the major pro-inflammatory cytokines implicated in inflammation-associated carcinogenesis, enhancing tumour cell growth [51,52]. Because the highest levels of IL-6 occurred at the onset of SCC induction, it is possible that this cytokine plays a role in cancer establishment in our model. IL-6 has also been shown to inhibit the extrinsic and intrinsic apoptotic pathways of skin cells, supporting the hypothesis that it may contribute to tumourigenesis [53]. Although IL-6 has previously been connected with squamous cell carcinoma bone invasion, which occurs during late stages of the disease [54], the highest concentration of this cytokine was detected at the beginning of DMBA-treatment. TNF-α also has also been proposed to contribute to squamous cell carcinoma tumour initiation and bone invasion [54] by stimulating the production of genotoxic molecules that can lead to DNA damage and mutations, such as NO [55], which is increased in all treated groups (Figure 2B) [56-58]. Levels of IL-13 were also diminished in chemically treated skin after the 4^th^ week (Figure 3C). Because IL-13 can negatively regulate anti-tumour immunity modulating NKT cell function, it may cooperate in cancer development [59].

The dual functions of IL-10 in antitumor immunity and immunoregulation have been recognized for some time [60]. In our study, the low levels of IL-10 detected in tumour initiation phase could be contributed to murine SCC development. IL-10 has been shown to modulate apoptosis and suppress angiogenesis and enhance the production of tumor-toxic molecules (e.g., nitric oxide) [61,62] and low levels of this cytokine could be favour tumor development. In fact, IL-10 deficient mice were more sensitive to DMBA/TPA induced papilloma [63]. In the promotion and progression phase, we detected a significant enhancement in IL-10 at 8 and 16 weeks after DMBA/TPA treatment. An IL-10 autocrine or paracrine loop might play an important role in tumour cell proliferation and survival through the upregulation of antiapoptotic genes such as BCL-2 or BCL-XL [64-66]. In addition, IL-10 inhibits secretion of the proinflammatory cytokines by CD4^+^ T cells and impairs CD8^+^ T cells response, whereas tumor clearance can be enhanced in the absence of IL-10 [67,68].

In the 16^th^ week, the cytokines IL-10, IL-17, TGF-β and TNF-α were detected at the highest overall level, creating a chronic inflammation cytokine milieu that may lead to antitumour immunity eradication and accelerated tumour progression. TGF-β enhances tumour invasion and, with TNF-α, affects stromal cells, facilitates angiogenesis, and impairs NK cells, CD8^+^ T cells and macrophage activity against tumours [58,69,70]. Additionally, TGF-β-induced inflammation in precancerous epidermal squamous lesions has been shown to require IL-17 [71]. IL-17 has also been associated with different types of cancer and may be expressed by tumour- associated macrophages and neutrophils to a lesser degree [69,72,73]. We found significant percentages of GR-1^+^ macrophages in the tumour tissue at 16 weeks (Figure 4B), and this macrophage phenotype has been reported to express IL-12p40 and iNOS [74]. However, GR1^+^F4/80^+^ cells have been reported to have negative effects on tumour protection [75]. Neutrophils and GR-1^-^ macrophages were the predominant cell type in lesion tissue at 4 and 8 weeks (Figure 4B), and GR-1^-^ macrophages are poor producers of NO [74]. These data suggest that MPO and NO were primarily produced by neutrophils at the start of SCC development and establishment (Table 2). However, ELA seemed to be primarily produced by activated macrophages along with IL-10 and IL-17, correlating with lesion appearance (Table 3).

In summary, the data presented here are in according with previous studies [2-9], which show that inflammatory mediators activate the remodeling of the tumor microenvironment through recruitment of leukocytes. The data presented here expand previous observation's by demonstrate that DMBA-induced inflammatory mediators are produced in the initial phase of carcinogenesis by activated neutrophils and macrophages. These findings may have broad implications besides providing a better insight into the mechanisms involved in DMBA-induced carcinogenesis. Increase of inflammatory mediators such as NO, active MPO and ELA, which are up-regulated in response to chronic inflammation, can increase mutation rates because induce DNA damage and genomic instability, in addition to enhancing the proliferation of mutated cells [2-9]. These events are associated with tumor initiation and progression, suggesting that inflammatory mediators may play an important role in initiation and promotion phase of SCC development. These findings represent a significant step towards in carcinogenesis.

## Conclusion

Our results suggest that activated neutrophils and macrophages are involved in inflammatory mediator production in tumour microenvironment. These cells may drive some immunity-related skin tissue damage and support cancer establishment.

## Competing interests

Authors declare no conflict of interest.

## Authors’ contributions

THG had the overall responsibilities of the experiment design and statistical analysis, the concept and wrote the manuscript. CdO carried out chemical induction of squamous cell carcinoma, histological experiments and counting of inflammatory infiltration in the lesions. LTdF carried out chemical induction of squamous cell carcinoma and counting of inflammatory infiltration in the lesions. CRP and RNR carried out chemical induction of squamous cell carcinoma. ALdScarried out histological experiments and counting of inflammatory infiltration in the lesions. GPG, JSdS and APC had shared the concept and supported the manuscript. APC had overall responsibilities of fund management, experimental design and wrote the manuscript. All the authors have read and approved the final manuscript.
